# Quality improvements in radiation oncology clinical trials

**DOI:** 10.3389/fonc.2023.1015596

**Published:** 2023-01-26

**Authors:** Koren Smith, Kenneth Ulin, Michael Knopp, Stephan Kry, Ying Xiao, Mark Rosen, Jeff Michalski, Matthew Iandoli, Fran Laurie, Jean Quigley, Heather Reifler, Juan Santiago, Kathleen Briggs, Shawn Kirby, Kate Schmitter, Fred Prior, Joel Saltz, Ashish Sharma, Maryann Bishop-Jodoin, Janaki Moni, M. Giulia Cicchetti, Thomas J. FitzGerald

**Affiliations:** ^1^ Imaging and Radiation Oncology Core-Rhode Island, Department of Radiation Oncology, UMass Chan Medical School, Lincoln, RI, United States; ^2^ Imaging and Radiation Oncology Core-Ohio, Department of Radiology, The Ohio State University, Columbus, OH, United States; ^3^ Imaging and Radiation Oncology Core-Houston, Division of Radiation Oncology, University of Texas, MD Anderson, Houston, TX, United States; ^4^ Imaging and Radiation Oncology Core Philadelphia, Department of Radiation Oncology, University of Pennsylvania, Philadelphia, PA, United States; ^5^ Imaging and Radiation Oncology Core Philadelphia, Department of Radiology, University of Pennsylvania, Philadelphia, PA, United States; ^6^ Department of Radiation Oncology, Washington University, St Louis, MO, United States; ^7^ Department of Biomedical Informatics, University of Arkansas, Little Rock, AR, United States; ^8^ Department of Biomedical Informatics, Stony Brook University, Stony Brook, NY, United States; ^9^ Department of Biomedical Informatics, Emory University, Atlanta, GA, United States

**Keywords:** oncology, clinical trials, patient care, quality, radiation therapy

## Abstract

Clinical trials have become the primary mechanism to validate process improvements in oncology clinical practice. Over the past two decades there have been considerable process improvements in the practice of radiation oncology within the structure of a modern department using advanced technology for patient care. Treatment planning is accomplished with volume definition including fusion of multiple series of diagnostic images into volumetric planning studies to optimize the definition of tumor and define the relationship of tumor to normal tissue. Daily treatment is validated by multiple tools of image guidance. Computer planning has been optimized and supported by the increasing use of artificial intelligence in treatment planning. Informatics technology has improved, and departments have become geographically transparent integrated through informatics bridges creating an economy of scale for the planning and execution of advanced technology radiation therapy. This serves to provide consistency in department habits and improve quality of patient care. Improvements in normal tissue sparing have further improved tolerance of treatment and allowed radiation oncologists to increase both daily and total dose to target. Radiation oncologists need to define *a priori* dose volume constraints to normal tissue as well as define how image guidance will be applied to each radiation treatment. These process improvements have enhanced the utility of radiation therapy in patient care and have made radiation therapy an attractive option for care in multiple primary disease settings. In this chapter we review how these changes have been applied to clinical practice and incorporated into clinical trials. We will discuss how the changes in clinical practice have improved the quality of clinical trials in radiation therapy. We will also identify what gaps remain and need to be addressed to offer further improvements in radiation oncology clinical trials and patient care.

## 1 Introduction

Radiation oncology has undergone significant change in patient care treatment processes during the past two decades. Modern radiation oncology training programs have limited resemblance to the programs designed by our mentors. Today, patients are planned with volumetric metrics with image fusion to optimize target definition based on the integration of multiple datasets including response adaptive radiation therapy (RT) planning. Multiple components of motion are managed through several pathways including four-dimensional treatment planning and optical tracking of patient positioning to provide security in daily treatment reproduction. With an increasing number of patients being treated with curative intent or re-treated with definitive fractionation, RT treatment planning is increasingly complex with the majority of patient treatments delivered with intensity modulation and advanced technology therapy techniques. In many clinical situations, higher daily doses are becoming more commonplace as our community becomes increasingly confident in our technology and reproducibility of daily treatment. Plans receive double checks by physics/dosimetry staff and often patients are imaged and not treated during the initial visit in order to confirm that all planning objectives are met, and the positioning is accurate. RT prescriptions include not only daily and total dose but now require a written strategy for dose, a document indicating normal tissue constraints and a written directive for how to apply daily image guidance. Smaller targets with normal tissue exclusion can be successfully treated with compressed fractionation and stereotactic techniques. Imaging tools incorporated into accelerator function serve as both an image positioning validation tool and can also function as a dosimeter confirming daily dose. These can be reviewed daily to ensure the quality and safety of each treatment. Because of the nimble nature of data acquisition through several informatics formats and the ability to display objects through multiple media, intra-departmental physician peer review of contours with image integration can be performed in real time. Therefore, data required for onsite peer review can be acquired and managed through facile onsite tool management strategies.

Many of these improvements are imbedded in protocols currently active in the National Cancer Institute (NCI) National Clinical Trials Network (NCTN) and other sponsored clinical trials and are applied at an enterprise level on a worldwide basis. Therefore, department processes used to manage quality and safety are re-purposed for quality management of clinical trials. In this chapter, the objective is to review how these process improvements are written into clinical trials and managed as part of the clinical trial data acquisition and management process and how these improvements ensure confidence in clinical trial outcome. Challenges remain with clinical trial processes as incomplete datasets and titration of data used to manage the clinical trial can often influence trial outcome. Issues likewise remain to be reconciled in trial management with real time adjustments in target location and how this should be managed in the context of the study. These improvements enhance the quality of treatment and improve patient care (1–17).

## 2 History of radiation oncology clinical trials

Although many disease areas in medicine do not have a structured mechanism for clinical trial function and management, oncology clinical trials have developed over the past half century with the NCTN. In the late 1960’s, seminal investigators began to design clinical trials in both adult and pediatric oncology to make progress in clinical care. RT was a nascent discipline, nevertheless important questions required consensus review including strategies for dose computation algorithms and radiation dose and fractionation for both tumor control and normal tissue tolerance. The Radiation Therapy Oncology Group (RTOG) developed clinical trials addressing these points in disease areas seminal to RT. The Gynecologic Oncology Group (GOG) integrated RT trials including brachytherapy (6, 14, 15). In multiple other cooperative groups RT would begin as an informal committee but developed voice as radiation was recognized as an important component to patient care. By the mid 1970’s investigators recognized the importance of data acquisition and review of treatment objects to ensure consistent application of RT to patients on trial. There was no established mechanism for this to occur, however early mentors in clinical trials imbedded data transfer strategies into the trials in order to establish a platform to review protocol compliance. The Quality Assurance Review Center (QARC) placed emphasis on data acquisition and data management including the collection of images to validate field placement for radiation oncology (18). QARC became an important feature in the clinical trials process as RT objects including dose computation, planning images and treatment portal images were collected in hard copy to be used for retrospective review of treatment for protocol compliance. QARC established a mechanism for on treatment review of objects whereas treatment plans could be reviewed as therapy was initiated in order to ensure the plan was compliant to study objectives. This significantly improved the quality of the trial as the study deviation rate was considerably improved by this process. QARC was complimented by the efforts of the Radiological Physics Center (RPC) (19–28). The RPC played an essential role in clinical trials by ensuring that dose computation and execution algorithms were compliant to standard (23). This was accomplished by multiple vehicles including the use of phantoms for treatment and review of RT planning dosimetry. Dose was measured by the RPC through multiple vehicles including thermoluminescence dosimetry (TLD) strategically placed in phantoms at critical locations. This would ensure that dose to both target and normal tissue was compliant to a national guideline. Because RT treatment planning was performed on fluoroscopy without the benefit of volumetric planning and validation imaging was performed with megavoltage, the perception in the first iteration of clinical trials was that quality of treatment was driven largely by the consistency of computational analytics and physics calibration. As time matured and volumetric imaging became available, imaging became an important vehicle to validate the target volume of interest and ensure that targets were treated in a protocol compliant manner and tumor was fully treated without exaggeration of dose to normal tissue. Up until this point however, there was no precise manner to validate specific dose to target from a volumetric perspective that could be trusted.

This changed with the development of RT digital planning tools. The Imaged Guided Therapy Quality Assurance (QA) Center became the leaders in this field and their effort and expertise brought RT clinical trials to a new level of performance (29). Prostate cancer became one of the first disease areas to be managed through this mechanism. The process of radiation treatment simulation for patients pivoted on this point and permanently changed for both the radiation clinic and clinical trials. Targets were drawn, slice by slice, on a planning computer tomography study. Normal tissues were contoured, and volumes of tumor and normal tissue were constructed. Radiation planning was now conducted through dose volumes and written directives were developed to specify dose to a volume. Constraints to normal tissue were applied to limit dose to a specified volume of normal tissue. Inverse planning tools for intensity modulation followed and fluence profiles within the beam could be modified to optimize both dose to tumor targets and limit dose to normal tissues. It was assumed, at this phase of development, that the imaging acquired for RT treatment planning was sufficient to determine protocol compliance. This major development permanently altered the approach to planning patients for RT as well as the management of clinical trials.

Jim Purdy and Jim Deye were essential contributors to the development of these tools, and both were kind to share these tools with QA centers. The cooperative groups associated with QARC, especially within the pediatric groups, had strong imaging committees and the RT committees had strong interest in the application of imaging to RT treatment planning. Although tools for image fusion of imaging objects into RT treatment plans had not yet matured at this time, images were collected at QARC for both assessment of response and validation of the targets chosen for RT. This became of particular importance in the early management of clinical protocols evaluating treatment strategies for Hodgkin lymphoma and have grown of increasing importance today as most clinical trials are now dependent on modern imaging to response assessment and outcome evaluation (30–43). In a series of early clinical trials involving patients of intermediate and high-risk disease, protocols were designed to test the utility of post chemotherapy RT including response adaptation of therapy in the mediastinum. In the Pediatric Oncology Group clinical trial 8725, imaging and RT objects were acquired as part of the data management process for the conduct of the study and reviewed at study closure. RT post-chemotherapy was the randomization point. For the entire study population, there appeared to be no benefit to the addition of RT to this patient population. However, because imaging and RT objects were onsite for secondary analysis, review of the data revealed that if patients received RT in a protocol compliant manner, there was a statistically significant improvement in survival and disease-free survival. The protocol had specific areas to target, and all disease sites noted at presentation had to be treated to protocol dose with appropriate dose titration to critical normal tissue structures including cardiac, pulmonary, and renal volumes. This implied that the quality of the application of RT had direct impact on patient and study outcomes and that images played an essential role in defining targets and assessing response. The challenge became moving this peer review process away from retrospective management and apply peer review in a more dedicated real time pre-therapy format in order to make certain trials were conducted in a protocol compliant manner. Pre-therapy review of imaging and RT objects in medulloblastoma clinical trial Children’s Oncology Group (COG) 9961 provided an unanticipated advantage of identifying patients who were not protocol eligible and assigned them to an appropriate study as those assigned to COG 9961 who were not eligible due to a higher stage and tumor burden had a statistically significant decrease in survival (44–50).

Pre-review of RT treatment objects was initially accomplished in early stage and intermediate stage Hodgkin lymphoma protocols COG 9425 and 9426. These studies included a component of response-adaptive therapy titration based on response to chemotherapy prior to the initiation of RT. Compliance to RT was significantly improved with the use of pre-review of objects, however, interestingly, there was a 50% discrepancy between central and site review of response to chemotherapy, therefore the need to review imaging objects for response as part of protocol management became the next process improvement in clinical trial management (45, 46).

The increasing use of digital data transfer permitted images to be acquired and reviewed by QA teams in a real time same-day format. This is important for trial management as it provided common ground review by site and study investigators as images could be reviewed by all involved in a simultaneous format and conflicts could be addressed and resolved before therapy could be applied. This was and remains important for adaptive studies. These studies are more complex to execute, and consistent interpretation of response ensures confidence in study conduct and outcome. Today, this is standard practice and cases from anywhere in the world can be reviewed on a same day basis for protocol management (45, 46, 51, 52).

Simultaneous real time review of imaging and RT objects as part of protocol management was accomplished in intermediate risk Hodgkin lymphoma study COG AHOD0031. This study was designed with an adaptive strategy with therapy titration including exclusion of RT in selected patients with initial rapid early response to chemotherapy and complete imaging response as defined by the study criteria. There was also a therapy augmentation component for patients who did not experience a rapid early response to initial chemotherapy. Therefore, consistent interpretation of response to therapy and application of RT treatment objects was essential for this trial which accrued nearly 1,800 patients. Review of anatomic and metabolic imaging by investigators was accomplished at several time points for each patient on study including at study entry for eligibility and after each two cycles of chemotherapy. RT treatment objects were reviewed pre therapy and outcome imaging was acquired per protocol. Outcome imaging has been an important component for this study and has generated many important publications including review of response to therapy for pleural effusions and bone. The study demonstrated that imaging datasets and RT objects could be managed and reviewed in a real time format in a manner identical to management of patients in a modern radiation oncology department (47, 48).

Because of the success in real time data management for clinical trials, this approach has been applied to most NCTN and other sponsored clinical trials and has been used in highly complex formats. For advanced stage Hodgkin lymphoma patients, RT is applied to areas of incomplete response or residual areas of disease measuring greater than 2.5 cm. For exceptionally young patients with minimal tumor burden removed by surgery, protocols include careful observation without additional therapy. Therefore, interpretation of imaging by study and site investigators is exceptionally important to ensure protocol objectives are met and the correct intended sites receive RT (51, 52).

Process improvements in RT with technology advancement, dose computation, image fusion, and clinical validation have been re-purposed and directly applied into NCTN and other sponsored RT clinical trials. In the next section, we will review how these improvements are applied in the daily workflow for the Imaging and Radiation Oncology Core (IROC). IROC serves as the imaging and radiation oncology core service for data acquisition and data management for the NCTN and other sponsored clinical trials. IROC credentials institutions and investigators for clinical trials and provides knowledge datasets to ensure that the sites can transfer the correct information to IROC. The knowledge tests often include contouring of tumor and normal tissues with generation of a dose plan in a protocol compliant manner.

## 3 QA process

As radiation oncology has matured with improved RT technology and image integration, the processes have matured and have been incorporated into the QA workflow. The segments of the program include site qualification, clinical trial design with support, site and individual credentialing, clinical trial management, case review, and support for trial analysis. The process is harmonized between multiple offices nationwide under a single integrated grant called the IROC. The office at IROC Houston manages site qualification and credentialing while the offices at IROC Rhode Island and IROC Philadelphia provide protocol case data acquisition and management with shared resources as needed for clinical trial support. The integrated informatics infrastructure has been provided by the American College of Radiology (ACR) and is called TRIAD (53).

## 4 Site qualification

Institutions and departments of radiation oncology can achieve recognition for being credentialed through multiple venues including but not limited to the American College of Radiology (ACR). Through a not dissimilar process, sites qualify for participation in clinical trials based on personnel, equipment, and the ability to apply technology according to the specific study. Generally, for protocol management, qualification includes completion of a questionnaire and validation of beam output for each unit treating clinical trial patients. This service verifies that a site has the basic resources and abilities to participate in NCI supported clinical trials. International sites must meet the same qualification requirements as North American sites. As of 2018, site qualification services are provided to 1,837 centers participating in clinical trials (including 140 international centers in 25 countries). The facility questionnaire is an electronic web-based form that is linked to the IROC database. This form collects information on the site’s demographics, staffing, treatment planning and delivery capabilities, and QA procedures. Since the facility questionnaire is linked to the IROC RT facility database, previous information regarding the site is filled in, requiring the site to simply add or modify existing information. Sites are required to review and edit their facility questionnaire annually. The questionnaire also collects information on RT imaging capabilities and supplies current information to the IROC roster used by the NCI Clinical Trial Support Unit (CTSU) and the Cancer Therapeutics Evaluation Program (CTEP). On an annual basis, all megavoltage photon beams, proton beams, and a selection of electron beams at every participating RT site have their reference beam output calibration measured remotely through a mailed TLD/optically stimulated luminescence dosimetry (OSLD) program to verify that beam output is within ±5%. High dose rate (HDR) (192Ir) brachytherapy and small field output factor OSLD/TLD remote audit tools have been developed and are in the initial stages of use (54). This output verification program is notable for its simplicity and would be similar to onsite QA measures performed by institutions as part of their internal program. When the TLD/OSLD measurement disagrees with an institution’s stated dose by more than 5%, IROC resolves this discrepancy through communication and procedural reviews. If those are unsuccessful, an on-site dosimetry visit may be performed.

Prior to a proton site enrolling patients on trials, they must be approved by IROC. In addition to the questionnaire and reference beam output verification, this process also includes successful completion of electronic transfer of proton treatment plans, irradiation of IROC’s baseline proton phantoms, and an on-site dosimetry review visit. Each proton delivery technique (passive scatter, intensity modulated proton therapy-(IMPT), etc.) used must be individually reviewed and approved.

## 5 Clinical trials support

Modern RT departments share or individually house the ability to generate clinical trials within the department or a trial generated by the cancer program involving RT. IROC offers expertise to help NCTN Groups develop new protocols, focusing on those sections relating to RT delivery, QA, and data collection. In a manner similar to an in-house data management group and Institutional Review Board (IRB), IROC reviews proposals at initial discussions including concept development through finalization of the protocol with efforts completed in a timely manner to not impede trial development. This early interaction is critical when new technologies or novel treatment techniques (e.g., developing QA strategies for radiopharmaceutical trials) are being introduced into trials. Because concept proposals typically lack detailed RT and imaging information, IROC provides a questionnaire to the concept Principal Investigator to gather the necessary information and facilitate further interaction as required. This in turn can help improve onsite investigator institutional trials mature with consistent treatment guidelines compliant to CTEP and support the conduct of the trial. After concept approval, IROC assists in the entire protocol development process. IROC contributes to developing and providing protocol RT section templates aimed to ensure quality trial data by standardizing structure name nomenclature, definition of dose volume analysis parameters, and radiotherapy processes in general. IROC’s protocol support includes but is not limited to 1) imaging procedures for target and organ at risk (OAR) definition, 2) dose prescription, 3) protocol compliance conditions, 4) RT treatment planning instructions; 5) QA procedures and their implementation, 6) image-guided RT (IGRT), and 7) the data submission process. By avoiding any unclear or ambiguous wording in the protocol and utilizing a consistent format, we ensure ease of understanding and implementation of the protocol.

## 6 Credentialing

Credentialing is the process of verifying that a specific site and/or clinician/physicist have the knowledge, resources, and capability to meet the protocol specifications. This process is analogous to independent peer review, and thus provides a basis for confidence in the institution and processes imbedded in the institution. Credentialing is distinct from ongoing periodic QA activities required for certification. Credentialing is designed and implemented through multiple pathways depending on the trial requirements. Credentialing may verify treatment planning, dose distributions, structure contouring, and/or image guidance. Credentialing tests may be systematic tests that assess the capabilities of the site or test the knowledge or skill of the investigator or planner. The success of credentialing is clear in that major deviation rates have decreased dramatically since credentialing has been required by protocols. While failure to meet the criteria may prevent a site’s participation in a specific protocol, the goal of IROC is not to restrict participation but to assist sites in any required remedial actions so that they meet the protocol criteria. Credentialing requirements are typically made generalizable so that if an institution successfully completes a credentialing requirement for one protocol, they do not need to repeat it for a subsequent protocol unless the new trial has a novel component. IROC has well established guidelines on when new credentialing is required and when an institution or investigator can be grandfathered through credentialing. To simplify the credentialing process, the presentation of protocol credentialing requirements has been condensed to a single table with links to the details of each requirement and implemented across all Groups. Additionally, an automated credentialing status inquiry system has been developed that maximizes clarity and ease of institutional inquiries and also provides credentials electronically to the CTSU’s Regulatory Support System (RSS) portal. Credentialing requirements may include completion of knowledge assessment/benchmark case or phantom irradiation depending on the specific protocol but are responsive to evolving protocol requirements including demonstrating successful site transfer of data to the QA centers. A meaningful minority of institutions still fail to meet the relatively lenient acceptability criteria. Importantly, these failures appear to be related to systematic problems at the institution, indicating that these errors will also affect protocol patients and potentially study outcomes. This highlights the importance of resolving any issues with the institution, and also highlights the value of this independent QA to supplement an institution’s internal QA program. Image guidance credentialing ensures that sites have an image guidance process that allows implementation of an appropriate imaging technique/image registration algorithm for the protocol and disease site. To minimize the burden on participating institutions, IROC has streamlined the IGRT credentialing to be based generically on bony registration or soft tissue registration. Institutions need therefore only pass two credentialing tests to be IGRT credentialed for all protocols (54–59).

## 7 Clinical trial management

The initial step in the QA evaluation process is review of the integrity of the submitted data (pre-review data management). This includes verifying that the institution has submitted accurate and complete protocol patient data to IROC. Incomplete and/or inaccurate files require additional IROC efforts and communication. To optimize the process, IROC has continual ongoing efforts to automate the QA of the data submission process. Clinical trials require that patients be treated as specified by the protocol. The purpose of case review is to verify that the sites planned and delivered the RT as required. A case review evaluates technical factors such as the dose distribution and fractionation and clinical factors such as the prescription, diagnostic imaging, tumor target/OAR contouring, and field placement. The case review service has been established so that NCI Groups have dedicated contacts within IROC for both imaging and RT. Standardizations for structure name nomenclature and dose volume parameter definitions are fully compliant with the American Association of Physicists in Medicine (AAPM) recommendations and templates for dose volume evaluation for different systems are published for each trial to aid institutions’ compliance. IROC has greatly increased the efficiency for dose volume analysis by creating the mechanism for automation of analysis and data upload. For institutional use, this would optimize individual case planning. For evaluation of submitted cases to IROC, scripts are developed for each trial to extract dose-volume histogram (DVH) data points and automatically format the data for direct upload to Medidata Rave (Rave), the NCI data management system. Institutions function in a similar manner with templates used for specification of image guidance and RT dose volume constraints. IROC has also implemented and developed knowledge-based tools and models to assess plan quality which can be re-purposed for institutional use. This has been done by evaluating and implementing knowledge-based systems/tools to assess the quality of RT treatment plans. These are commonly used for NCTN, and other sponsored trials (60).

There are three types of case review during the active period of the protocol: Pre, On, and Post-treatment review. These differ in the time when they are initiated relative to the start of patient treatment. All reviews evaluate the cases for protocol compliance. IROC will evaluate the effectiveness of case reviews using the large amount of information relating to institution performance in complying with protocols. Pretreatment review occurs prior to the start of treatment so cases can be modified to meet protocol objectives. The challenge of this review is that it requires coordinated timing to have the case reviewed promptly by IROC to avoid delay of the start of the patient’s treatment. Pre-treatment reviews also provide an interactive forum between study and site investigators. This process helps to determine if protocol amendments are needed. For institutions, this process resembles goals and objectives achieved in chart rounds and peer review. The on-treatment review is performed within seven days from the start of RT and results are communicated to the site for the purpose of improving overall treatment. Post treatment review records the details of the treatment given (including changes that are made after the initial review). The process evaluates technical aspects as well as verification that the RT data elements captured are accurate. Upon completion of case accrual for a trial, or for interim analysis, IROC provides information as requested by the NCTN Group for analysis. This process is similar to processes performed by institutions for quality metrics or publication.

## 8 Data management

IROC is responsible for holding the Digital Imaging and Communications in Medicine (DICOM) imaging and RT data for all NCTN Group clinical trials that use RT for treatment. These datasets are cross-referenced to the case data entered into the NCI information system RAVE. These data are used for protocol endpoint analysis including tools to obtain crucial data points from dose volume submissions not included in the initial design of the protocol. IROC is the custodian of the data on behalf of the NCI Groups, and use of the data will be determined by the NCI and the NCTN data resource sharing plan for each group. IROC can support data analyses by 1) gathering, packaging, and forwarding de-identified data to the requesting organization or group meeting 2) providing analysis relating to dose distributions and structure contouring for secondary analysis requests 3) providing services relating to tumor response and critical structure complications modeling and 4) provide data to support reviews not anticipated at the time of study initiation. IROC may also use these data directly for the purpose of quality improvement and effectiveness research (14, 15, 54–69).

## 9 Conclusion

The RT QA processes and procedures offered by IROC are diverse, dynamic and subject to continuous review and adaptation, similar to process improvements seen in daily department function. IROC endeavors to ensure that the QA services are as efficient and effective as possible in order to reasonably handle the clinical trial workload from NCI Group studies and industry trials. The systems developed, data collected, and services provided by IROC offer opportunities for collaborations with the NCI Groups, both nationally and internationally. As a service organization, IROC achieves the highest quality patient data allowing the NCI to meet its clinical trial goals. This is important for primary trial analysis as well as analysis of both secondary and tertiary trial objectives. A complete and accurate dataset increases the likelihood the outcome can be trusted and moved into clinical practice. Processes in the QA of clinical trials can be re-purposed by individual institutions through real time peer review and ensure compliance to standard between colleagues. Many tools exist through multiple vendors which can display image objects and radiation targets during a chart rounds session which can mimic the process of real time review for clinical trial activity. There are multiple checks for the computational component of our work and chart completeness. Coupling this effort with physician peer review will harmonize activity within a department and bring the physicians closer to the departmental QA process and into compliance standards for regulatory review. Research platforms can be built through this prism.

Each patient treated with RT can be part of a clinical trial. The trials can be investigator driven with endpoints following many pathways including outcome analysis, normal tissue tolerance, patient support, physics, and nursing. To do so at an enterprise manner would require infrastructure similar to The Cancer Imaging Archive (TCIA) imbedded into daily department activities and workflow with transfer of onsite objects to TCIA as seen appropriate by the TCIA steering group. The information technology infrastructure of TCIA is PRISM and this platform houses patient objects including pathology/pathomics, radiology, RT treatment objects, medical oncology information, and relevant clinical information. All of this information can be re-purposed for modern projects including but not limited to artificial intelligence. Many departments now house TCIA informatics infrastructure as part of internal data management services to use on a daily basis for translational research. As data including pathology evolves in digital format, research can be completed with nimble query tools to help move our translational science forward (66–77).

The future of our discipline is bright. We have to be disciplined in our science place emphasis on the quality of our data. If we do so, our work will be well perceived and colleagues can trust our results.

## Author contributions

All authors listed have made a substantial, direct, and intellectual contribution to the work and approved it for publication.
